# Dynamic SLAM by Combining Rigid Feature Point Set Modeling and YOLO

**DOI:** 10.3390/s26010235

**Published:** 2025-12-30

**Authors:** Pengchao Ding, Weidong Wang, Xian Wu, Kangle Xu, Dongmei Wu, Zhijiang Du

**Affiliations:** State Key Laboratory of Robotics and Systems, Harbin University of Technology, Harbin 150001, China; dpc950220@163.com (P.D.); shanson163@163.com (X.W.); 25s108201@stu.hit.edu.cn (K.X.); wdm@hit.edu.cn (D.W.); duzj01@hit.edu.cn (Z.D.)

**Keywords:** dynamic SLAM, visual SLAM, object detection

## Abstract

**Highlights:**

**What are the main findings?**

- This paper combines target recognition and depth threshold segmentation to rapidly segment the target point cloud
- This paper integrates Kalman filtering and the depth intersection-over-union method (DIoU) for the association of target bounding boxes.
- This paper proposes the adaptive Rigid Point Set Modeling and creates rigid and non-rigid factors for the factor graph optimization in the SLAM system.

**What are the implications of the main findings?**

- Achieve real-time screening of dynamic feature points.
- When occlusions occur among dynamic targets, the system can still effectively track the targets.
- Improve the positioning accuracy of the SLAM system in dynamic environments.

**Abstract:**

To obtain accurate location information in dynamic environments, we propose a dynamic visual–inertial SLAM algorithm that can operate in real-time. In this paper, we combine the YOLO-V5 algorithm and the depth threshold extraction algorithm to achieve real-time pixel-level segmentation of objects. Meanwhile, to address the situation where dynamic targets are occluded by other objects, we design the object depth extraction method based on K-means clustering. We also design a factor graph optimization with rigid and non-rigid dynamic objects based on object category division, in order to better utilize the motion information of dynamic objects. We use the Kalman filter algorithm to achieve object matching and tracking. At the same time, to obtain as many rigid targets as possible, we design the adaptive rigid point set modeling algorithm to further supplement the rigid objects. Finally, we evaluate the algorithm through public datasets and self-built datasets, verifying its ability to handle dynamic environments.

## 1. Introduction

SLAM technology plays an important role in the field of autonomous robots. It allows robots to perceive and understand their environment in real-time without relying on external positioning systems, thus enabling efficient movement and task execution. In the SLAM system, the assumptions are as follows: features in the environment are stationary and recognizable; there is a definite relationship between sensor observations and environmental features; the robot’s motion conforms to the kinematic model; and both motion and noise follow a normal distribution. Based on these basic assumptions, the SLAM system obtains the mapping relationship between poses and features from multiple frames of data, constructs an error function, and then minimizes the error function through algorithms such as graph optimization, thereby obtaining the robot’s positioning information and feature map. However, as the robot’s working environment gradually shifts to more complex environments, dynamic objects such as moving people and vehicles clearly violate the assumption that features in the environment are stationary, causing the system’s positioning to become unstable.

This paper assumes that the motion of dynamic people or objects also conforms to the kinematic model, and their motion is considered estimable. By adding motion information to dynamic features based on the motion model, this paper achieves a kind of “quasi-static” state, thus enabling the inclusion of these features in the graph optimization algorithm. We propose a new dynamic SLAM method in this paper. The method combines object detection, motion tracking, and factor graph optimization to effectively handle occluded moving objects. Specifically, we propose an algorithm for obtaining target depth based on K-means, which effectively acquires the depth of occluded objects. Then, based on the depth of the background and occluding objects, we perform depth threshold segmentation on the image to obtain the corresponding mask for the target. Next, we design a Kalman filter that incorporates depth information to effectively track objects. Finally, we add dynamic features to factor graph optimization for robot real-time localization, based on rigid and non-rigid categories.

We conducted experiments in different scenarios and compared them with traditional SLAM methods. The experimental results show that our dynamic SLAM method can handle moving objects with mutual occlusion, achieving motion estimation. Compared with traditional methods, our method has a significant improvement in the precision of motion estimation.

The following are the primary contributions of this paper to the aforementioned situation:

(1) We combine deep threshold based on YOLOv5, K-means clustering, and overlapping masks to effectively separate potentially moving objects, including moving objects with mutual occlusion.
(2) We use a Kalman filter to predict target bounding boxes that contain depth information and use the DIoU method to match predicted and detected bounding boxes.
(3) We propose adaptive rigid point set modeling to extract rigid feature points from non-rigid objects.
(4) We propose a factor graph optimization-based SLAM that includes factors related to both rigid and non-rigid objects.

Besides the introduction, the components of this paper are as follows. Section 1 introduces the relevant work. Section 2 describes the overall SLAM scheme and basic issue from the moving objects with mutual occlusion. Section 3 includes four parts: (1) object segmentation, (2) object tracking, (3) adaptive rigid point set modeling, (4) factor graph optimization for dynamic objects. Section 4 presents the results of practical experiments, and Section 5 offers some concluding remarks.

## 2. Related Work

Achieving good perception of the environment and understanding its own position has always been an important research direction in the field of robotics. In order to obtain accurate positioning information from camera images, numerous scholars have conducted related work. Mourikis proposed two frameworks, MSCKF [1] and MSCKF2.0 [2], based on the Extended Kalman Filter, an established measurement model to use static feature points as constraints. Meanwhile, many scholars have also conducted research based on optimization to solve the problem of the Kalman Filter’s reduced accuracy. Qin proposed VINS-mono [3] and VINS-Fusion [4] to achieve complete visual–inertial SLAM. R. Mur-Art proposed three versions of ORB–SLAM [5,6,7] using multi-threaded methods, which greatly improved the positioning accuracy.

However, when the above methods are applied in environments with dynamic objects, their positioning accuracy decreases. To address this issue, scholars have conducted dynamic detection of feature points, for example, improved RANSAC [8]. However, its accuracy is still relatively low.

With the rapid development of deep learning methods, scholars have also started to consider combining the deep learning algorithms emerging in the fields of object recognition [9,10,11] and segmentation [12,13]. Chao Yu [14] used the SegNet segmentation network and the RANSAC method to remove dynamic points, but it can only handle a small number of dynamic features and is time-consuming. Berta Bescos combined Mask R-Cnn to propose DynaSLAM [15], which removes dynamic features through disparity and depth differences between frames, but it still takes a long time. In order to alleviate the dependence of deep learning on hardware and ensure the real-time performance of the system, recent scholars have removed dynamic features by combining object detection algorithms with dynamic detection algorithms. YDD–SLAM [16], YPR–SLAM [17], and DI–SLAM [18] introduce the YOLO-v5 algorithm for potential target recognition. The difference lies in the strategies for discriminating dynamic features. YPR–SLAM uses the depth fluctuation threshold, while DI–SLAM uses the parallax angle threshold.

Unlike the method of directly removing dynamic points mentioned above, scholars have gradually incorporated the motion of the objects into the optimization framework to better utilize the information in dynamic features. Jun Zhang proposed VDO–SLAM [19], which uses Mask-RGBRCNN for image segmentation, PWC–Net for computing dense optical flow and incorporates the motion of rigid objects into the optimization of the SLAM backend. However, too many deep networks make the framework difficult to run in real-time. Based on DynaSLAM, Berta Bescos proposed DynaSLAM2 [20], which uses 2D instances for matching dynamic objects. However, both assume that objects are rigid, so the localization accuracy decreases when dealing with non-rigid objects. Therefore, Yuheng Qiu proposed AirDos [21], which uses the Alpha-Net network to model the object as a hinge-like object, while computing dense optical flow using PWC–Net. However, it still takes a long time.

Due to the scenes containing many dynamic objects and the requirement of real-time operation of robots, we propose an algorithm to improve the positioning accuracy without causing excessive time consumption.

## 3. System Overview

Firstly, we define the frames and state variables used in this paper. We define the world frame as **W**, the camera frame as **C**, and the IMU frame as **I**. We take the camera frame as the frame for our robot **R**. The goal of this article is to estimate the robot’s position relative to a fixed world frame. Thus, we define the robot’s initial position as the world frame. Then, we define the state variable $X_{k}$ of the robot, as shown in Equation (1).

(1) $$X_{i}=\left\{P_{i},V_{i},O_{i},H_{i},M_{i},N_{i},b_{i},d_{i}\right\},i\in \left(0,n\right)$$

where the ith state of the robot includes the position $P_{i}$, the velocity $V_{i}$, the orientation $O_{i}$, features of rigid motion objects $H_{i}$, features of non-rigid motion objects $M_{i}$, static features $N_{i}$, accelerometer bias $b_{i}$, and gyroscope bias $d_{i}$.

When a rigid object moves, the relative positions of the feature points on its body remain unchanged. This means that after estimating the object’s motion, the motion of its feature points can be obtained through relative motion. Conversely, for non-rigid objects, the relative positions of the feature points on their bodies are not fixed, so we need to process each feature point individually. For example, a moving car can be considered a rigid object, while a person is a non-rigid object.

**Figure 1:**
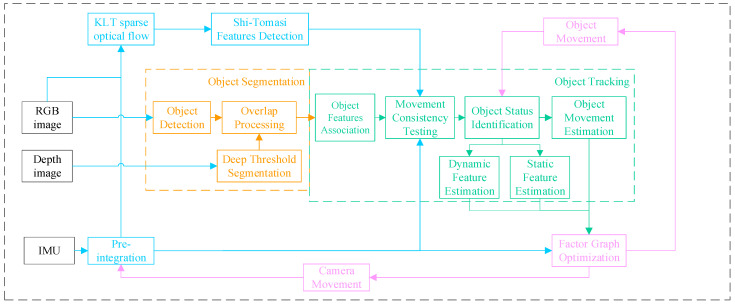
Block diagram illustrating the full pipeline of the propose dynamic SLAM. The pipeline is described in Section 3, the object segmentation is described in Section 4.1 and the object tracking is described in Section 4.2.

The SLAM system proposed in this article uses RGB-D cameras and IMU sensors, and the overall algorithm framework is shown in Figure 1. The algorithm includes three parts: object segmentation, object tracking, and visual SLAM based on optical flow.

**Figure 2:**
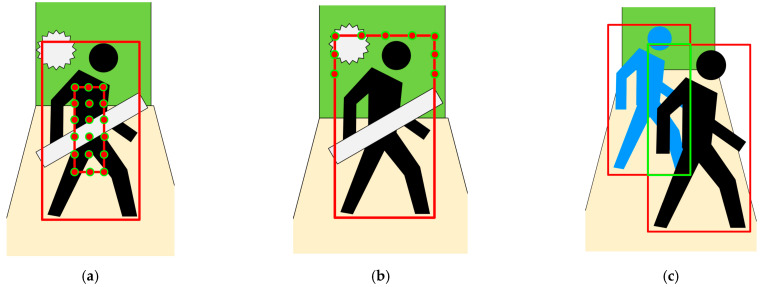
Object segmentation diagram: The yellow part represents the ground, the green part represents the wall at the end of the corridor, the gray rectangle and polygon represent obstructing objects, the larger red box represents the YOLO detection box, the green box represents the overlapping part of the YOLO detection boxes, and the solid red circle represents the candidate points. (**a**) k-means selects the depth of the target, (**b**) obtain the background depth. (**c**) mutual occlusion.

The SLAM structure in this article mainly consists of two parts: feature extraction and tracking, and camera pose optimization based on factor graphs. After receiving RGB-D images, we divide the processing into two parts: (1) object segmentation module for object recognition and segmentation, obtaining the corresponding region of each object in the image, as shown in the orange part of Figure 1; (2) Feature extraction module, first extracting Shi-Tomasi features, and then using KL optical flow to match inter-frame feature points based on the camera motion obtained by IMU pre-integration, as shown in the blue part of Figure 1. After obtaining the region of the object and the matching point pairs in the image, the object tracking module provides the matching of the objects between images.

And then, according to motion consistency analysis and object type, the feature points are divided into three types: static features, rigid object features, and non-rigid object features, corresponding to the green part of Figure 1. For rigid objects, we estimate the object’s motion and then associate each feature point with it based on relative position. For non-rigid features, we use a constant velocity model to estimate the motion of each dynamic feature separately. For static features, we use reprojection to estimate their positions in the image. Finally, we establish a factor graph to optimize the camera pose and object motion, as shown in the pink part of Figure 1. Finally, the obtained camera motion and object motion are used as initial values to participate in IMU pre-integration and object motion estimation respectively.

## 4. Methodology

The SLAM algorithm described in this article is mainly aimed at scenes containing moving objects and where the moving objects are occluded. Here we mainly introduce the following three parts: Object Segmentation, Object Tracking, and Factor Graph.

### 4.1. Object Segmentation Process

To address the poor real-time performance of mainstream segmentation algorithms and the low accuracy of object detection algorithms, this paper combines object detection and deep threshold segmentation to achieve real-time object segmentation.

In the depth image, the background corresponds to the distant part behind the object, and the occlusion corresponds to the closer part in front of the object. Therefore, a threshold method can be used for quick filtering.

For object detection, we use YOLO-v5 [11], which is an object detection algorithm that uses deep learning and convolutional neural network to obtain bounding boxes for multiple objects in an image, as shown in Figure 2a–c. As the main dynamic objects in the environment are people and cars, with a low proportion of other objects, we use this algorithm for detecting these two types of objects.

Due to the presence of obstructing objects, we use a method of measuring the object’s depth by multiple candidate points, as shown by the red solid circle in Figure 2a. Firstly, the candidate box *M* is obtained by changing the YOLO detection box so that the center position of the YOLO detection box remains stable, and the size is reduced by half. Then, we select *m* points evenly on the candidate box to obtain depth values, then use the K-means [22] algorithm to cluster the *m* candidate points into two categories. The average depth value of the points in the category with greater depth is the object’s depth value $d_{t}$. The average depth value of the smaller category is the occlusion’s depth value $d_{o}$. Then, the small depth threshold can be obtained from Equation (2).

(2) $$T_{\min}=d_{t}/2+d_{o}/4$$

The lower part usually represents the ground, which cannot effectively reflect the depth of the distant part, as shown in Figure 2b. Therefore, when obtaining frame *k*, we uniformly sample *n* points *P* from the upper part of the frame. Then we use the three-sigma rule of thumb to remove the outliers generated from occlusions, as shown in Figure 2b. The average depth value of remaining points is the background’s depth value $d_{b}$. The large depth threshold can be got from Equation (3).

(3) $$T_{\max}=\left\{\begin{array}{cc} d_{t}/2+d_{b}/2 & \left(d_{b}-d_{t}\right)>e\\ d_{t}+e & else \end{array}\right.$$

where *e* is a predefined value (0.5 for people and three for cars).

After obtaining two depth values, we perform threshold segmentation, in which the part of image with depth values between $T_{\min}$ and $T_{\max}$ will be saved.

In addition, different targets also obstruct each other, as shown in Figure 2c. For this situation, we use the same method as the above method.

### 4.2. Object Tracking Process

**Figure 3:**
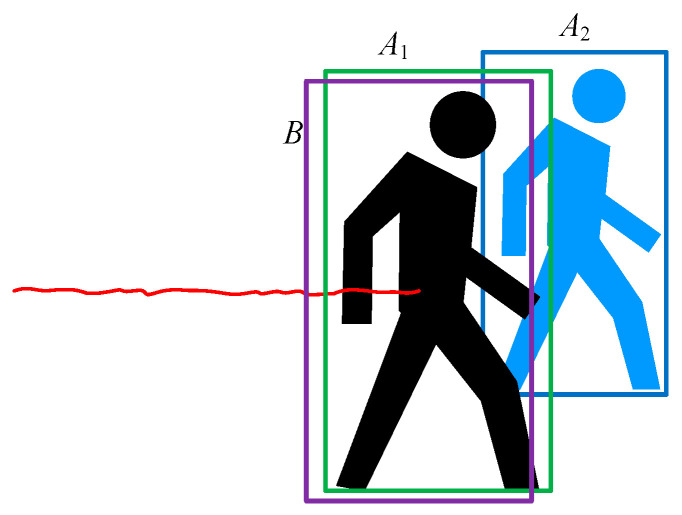
Schematic Diagram of Prediction and Matching: The red curve represents the historical trajectory. The box labeled A1 or A2 is the YOLO-recognized box in the current frame, and the purple box labeled B is the predicted box of the currently tracked target.

In order to associate each object detected by YOLO in different frame images, this paper uses the Kalman filtering method to track each object, as shown in Figure 3. Through prediction, we can effectively deal with the phenomenon of overlapping and occlusion of recognition boxes caused by object movement.

This paper assumes that dynamic objects move continuously in space, meaning that their corresponding YOLO recognition boxes change continuously, and the corresponding depth changes are also continuous. Therefore, by combining the observation data (recognition boxes with depth) of each frame with the motion equation, the target box of the next frame can be predicted. Here, this paper uses the Kalman filter algorithm to predict the target box. The observation variables in the Kalman filter include: the center position (*u*,*v*) of the target box, the bounding box area *s*, and the depth information *d* of the target. Here, we assume that the aspect ratio of the box remains constant for a short period of time. Then, the state variables in the Kalman filter include: observation variables u,v,s,d, the rate of change of observation variables $\dot{u},\dot{v},\dot{s},\dot{d}$, and the aspect ratio *r*. Therefore, the state can be represented as Equation (4), and the measurement can be represented as Equation (5).

(4) $$X=\left[u,v,s,d,r,\dot{u},\dot{v},\dot{s},\dot{d}\right]$$

(5) $$Z=\left[u,v,s,d\right]$$

When the posterior state ${\hat{x}}_{k-1}$ and the covariance ${\hat{P}}_{k-1}$ of the posterior probability distribution of the previous frame are obtained, the prior state variables ${\overset{⌣}{X}}_{k}$ and covariance ${\overset{⌣}{P}}_{k}$ of the *k*-th frame are as shown in Equation (6).

(6) $${\overset{⌣}{X}}_{k}=A_{k}{\hat{X}}_{k-1},\;{\overset{⌣}{P}}_{k}=A_{k}{\hat{P}}_{k-1}A_{k}^{T}+R$$

where *R* is the covariance matrix of measurement noise, and *A* is the state transition matrix, as shown in Equation (7).

(7) $$A=\left[I_{9\times 9}\right]+\left[\begin{array}{cc} 0 & \delta tI_{4\times 4}\\ 0 & 0 \end{array}\right]$$

After obtaining the measurement values, we update the filter, where the Kalman gain *K* is shown in Equation (8).

(8) $$K={\overset{⌣}{P}}_{k}C_{k}^{T}{\left(C_{k}{\overset{⌣}{P}}_{k}C_{k}^{T}+Q_{k}\right)}^{-1}$$

where the observation matrix is $C=\left[\begin{array}{cc} I_{5\times 5} & 0_{5\times 4} \end{array}\right]$ and *Q* is the covariance matrix of the process noise.

At this point, through YOLO, we obtain the target box *A* in the *k*-th frame. By analyzing the parameters of ${\overset{⌣}{X}}_{k}$, we can obtain the target box *B*. We use the depth intersection-over-union method (DIoU), as shown in Equation (9), to calculate the weights of predicted bounding boxes and obtained bounding boxes, and then choose the best match based on the weights.

(9) $$\rho =\frac{S_{A\cap B}d_{\max}}{S_{A\cup B}\left|d_{A}-d_{B}\right|}$$

where *A* and *B* are the regions of predicted and detected bounding boxes in the image, and $d_{A}$ and $d_{B}$ are their corresponding depths. Meanwhile, if ρ is greater than 0.7, it will be considered a successful match, otherwise the object is deemed not present in the image.

After a successful match, we assign the parameters of the YOLO detection box corresponding to the biggest *DIoU* as the measurement value to $Z_{k}$ in Equation (10).

(10) $${\hat{X}}_{k}={\overset{⌣}{X}}_{k}+K\left(Z_{k}-C_{k}{\overset{⌣}{X}}_{k}\right),\;{\hat{P}}_{k}=\left(I-KC_{k}\right){\overset{⌣}{P}}_{k}$$

By Equation (10), we get the posterior state ${\hat{X}}_{k}$ and the covariance ${\hat{P}}_{k}$ of the posterior probability distribution of the current frame. We then predict the target box of the next frame, and enter the next cycle of prediction–matching–updating–prediction.

### 4.3. Adaptive Rigid Point Set Modeling

This paper classifies dynamic targets into rigid objects and non-rigid objects. Although all feature points on a rigid object are in motion, the relative positional relationship between them remains almost unchanged. When analyzing the rigid objects, all dynamic points on them can be treated as a single unit for analysis. In contrast, the relative positional relationship between the various feature points on a non-rigid object does not remain invariant during movement. However, for the human body, as a kind of non-rigid object, which is the research object of this paper, the dynamic feature points within the torso can be regarded as moving rigidly. Therefore, this paper designs a dynamic target modeling strategy for adaptive rigid bodies.

This paper adopts the field flow method to evaluate the movement of feature points. Assuming that the coordinates of a feature point in the camera coordinate system at the *k*-th frame are denoted as $P_{k}\left(x_{k}^{c_{k}},\;y_{k}^{c_{k}},\;z_{k}^{c_{k}}\right)$, and its coordinates in the camera coordinate system at the (*k* + 1)th frame are denoted as $P_{k+1}\left(x_{k+1}^{c_{k+1}},\;y_{k+1}^{c_{k+1}},\;z_{k+1}^{c_{k+1}}\right)$, the field flow of the feature point is calculated using Equation (11). When the average norm of the field flows of all feature points in the point set exceeds the threshold, the target is considered a dynamic object.

(11) $$F=P_{k+1}-T_{w}^{c_{k+1}}T_{c_{k}}^{w}P_{k}$$

**Figure 4:**
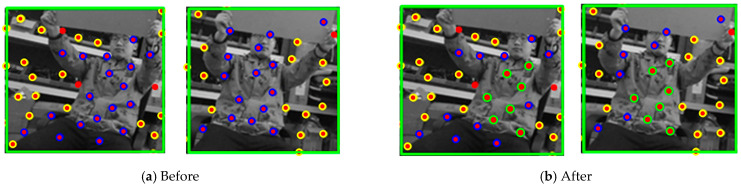
Schematic diagram of rigid feature point set extraction. The yellow colored dots represent static feature points, the blue colored dots denote dynamic feature points and the green colored dots denote the rigid feature point set.

As a typical non-rigid object, the human body still has some feature points whose relative motion remains unchanged, such as the feature points within the green circles shown in Figure 4b. Evaluating these points motion by treating them as an integral whole can effectively reduce noise interference. Assuming that we obtain the set η of matched feature points from the two frames of images illustrated in Figure 4a, where the points in the previous frame are denoted as $p_{i}$, and the point cloud in the subsequent frame is denoted as $q_{i}$, the process of extracting the rigid feature points from the set is as follows, which is the coarse registration process:

1. Randomly select four pairs of corresponding points. Calculate the centroid-removed coordinates by Equation (12), compute the rotation matrix *R* by Equation (13), and calculate the translation matrix *t* by Equation (14), where *n* is set to 4.
  (12) $${p\prime }_{i}=p_{i}-\frac{1}{4}\sum_{k=1}^{n}p_{k},{q\prime }_{j}=q_{j}-\frac{1}{4}\sum_{k=1}^{n}q_{k}$$
  (13) $$R=\underset{R}{\arg\min}\frac{1}{2}\sum_{k=1}^{n}\left\|{q\prime }_{k}-R{p\prime }_{k}\right\|$$
  (14) $$t=\frac{1}{4}\left(\sum_{k=1}^{n}q_{k}-R\sum_{k=1}^{n}p_{k}\right)$$
2. We calculate the transformed position ${p\prime }_{i}$ of each $p_{i}$ by using Equation (15), and then compute the Euclidean distance between ${p\prime }_{i}$ and $q_{i}$. If the distance is less than the threshold, the point is regarded as an inlier, and then we count the total number *N* of inliers.
  (15) q=Rp+t
3. To obtain the inlier set with the maximum number of inliers, repeat Steps 1 and 2 until 100 iterations are reached.

**Figure 5:**
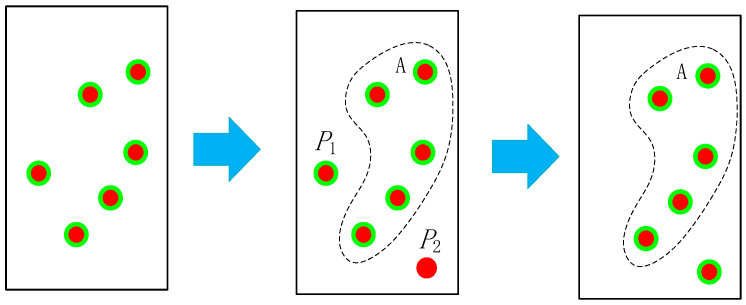
Schematic diagram of the disappearance and addition process of rigid feature point sets. The green colored dots denote dynamic feature points, the point set enclosed by the dotted line is a stable rigid point set, *P*_1_ represents the dynamic feature point that is about to break away from the rigid point set, *P*_2_ represents the newly added dynamic feature point.

Through the above steps, we obtain the rigid feature point set existing on the human body. We then calculate the optimal transformation matrix (*R_b_*, *t_b_*) for all inliers using Equations (13) and (14), which is the precise registration process. However, when the target moves, the rigid feature points will gradually increase or disappear, as shown in Figure 5. Point *P*_1_ disappears, and the new matching Point *P*_2_ appears in the third frame. Therefore, we design a point set update strategy.

After a new rigid point set is established, we use this centroid as the virtual centroid *p*. For all subsequent calculations of the translation matrix, the virtual centroid shall be adopted instead of the actual centroid. We calculate the position of the virtual centroid of the current frame based on the matched point pairs, then use the coarse registration process to determine whether new inliers are added. If there are new inliers, we perform the precise registration to compute the transformation matrix.

Up to this point, we have obtained the motion state of the rigid feature point set. The process of acquiring the motion of a rigid body is similar to this one.

### 4.4. Factor Graph with Non-Rigid and Rigid Feature Points

**Figure 6:**
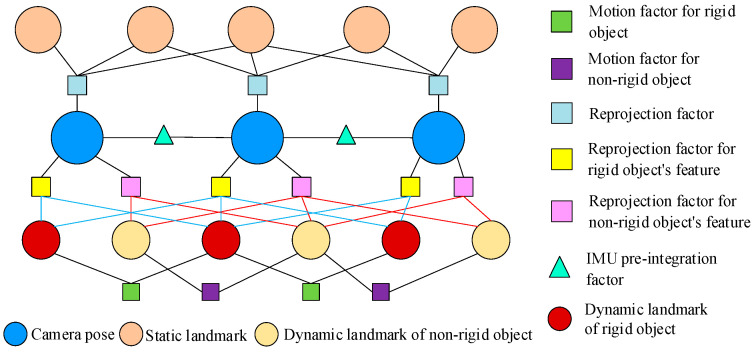
Factor graph.

For static feature points, the method used in this paper is the same as in Vins-fusion [3]: extracting Shi–Tomasi feature points, grid-based uniform extraction, and LK optical flow tracking based on imu pre-integration. Like Vins-fusion [3], the optimization part also includes IMU pre-integration factor $r_{b}\left(Z_{b_{n}b_{n+1}},X\right)$, reprojection factor $r_{\lambda }\left(Z_{\lambda_{m}}^{c_{n}},X\right)$, and sliding window marginalization factor $r_{p}-J_{p}X$. With these, we obtain multiple pairs of feature points by tracking features, and obtain the corresponding camera poses for each frame image through factor graph optimization. However, dynamic features do not comply with the assumption of map invariance in Vins-fusion, and their proximity to the camera can introduce significant biases, leading to system crashes. Therefore, we introduce dynamic feature smooth motion constraints $r_{D}\left({\hat{z}}_{d}^{c_{n}},x\right)$, dynamic feature reprojection constraints $r_{O}\left({\hat{z}}_{k}^{c_{n}},x\right)$, and dynamic object smooth constraints for different dynamic feature points romHn−1n−2lW,Hnn−1lW. The system factor graph is shown in Figure 6. The optimal state estimation is obtained by minimizing the objective function, as shown in Equation (16).

(16) argminxρrp−JpX∑p2+∑n,m∈λρrλZλmcn,X∑λmcn2+∑n,d∈DρrDz^dcn,x∑Ddcn2+∑n,k∈DρrOz^kcn,x∑Dkcn2∑n,l∈omρromHn−1n−2lW,Hnn−1lW∑lcn2+∑n∈BρrbZbnbn+1,X∑bnbn+12

Dynamic feature smoothing motion constraints are used to constrain non-rigid dynamic feature points. The relative positional relationships between feature points on non-rigid targets often change, so they need to be considered separately. In this paper we consider people as the object, and the motion range of feature points on their bodies is small. Therefore, for this type of feature, the constant velocity model is used to simplify the constraints, as shown in Equation (17).

(17) $$r_{D}\left({\hat{z}}_{d}^{c_{n}},x\right)=p_{d}^{c_{n}}-\pi_{c}\left(T_{c}^{b}T_{b_{n}}^{w}p_{d}^{w_{n}}\right)$$

where $\pi_{c}$ is the camera projection function, $T_{b_{n}}^{w}$ is the transformation from the IMU coordinate system *b_n_* of the *n*th frame to the world coordinate system ***W***. $p_{d}^{w_{n}}$ is the predicted position of the *d*th dynamic feature in the world coordinate system. $p_{d}^{c_{n}}$ is the observed position of the feature in pixel coordinates. $T_{c}^{b}$ is the transformation from the camera coordinate system to the IMU coordinate system.

For rigid objects or rigid points set, the relative positions of their various parts do not change. Therefore, we can use their motion to construct re-projection constraints for dynamic features. Here we use homogeneous transformation Hnn−1W to represent the motion of the target from the (*n* − 1)th frame to the *n*th frame, and use LnW to represent the 3D pose of the object in the *n*th frame to the world coordinate system. Then,

(18) Hnn−1W=Ln−1WLnW

Thus, for the *k*th feature point on the object in this continuous motion, we can predict its position m^nkw in the world coordinate system, as shown in Equation (19).

(19) m^nkw=LnwHn−1nWLn−1−1wmn−1kw

Similar to the reprojection residual of static features, we can establish Equation (20) to optimize the object motion and camera pose.

(20) rOz^kcn,x=pkcn−πcTcbTbnwm^nkw

where $p_{k}^{c_{n}}$ is the position of the feature point in the image.

For rigid objects, we also establish smooth motion constraints as shown in Equation (21).

(21) romHlW,n−1n−2Hnn−1lW=Hn−1n−2lWHnn−1lW

## 5. Experiments

**Figure 7:**
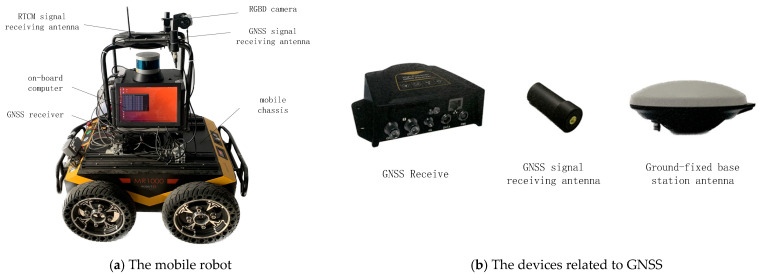
The mobile robot configuration diagram.

In this section, we conduct experiments related to deep threshold segmentation and tracking to evaluate the effectiveness and real-time performance of the algorithm. Then, we evaluated the ability of the SLAM algorithm to handle dynamic objects in both datasets and real-world experiments. The mobile robot consists of intel Intel RealSense D455 RGB-D camera (Intel Corporation, Santa Clara, CA, USA), M66-Lite GNSS receiver, mobile chassis, and on-board computer, as shown in Figure 7.

### 5.1. Object Segmentation

**Figure 8:**
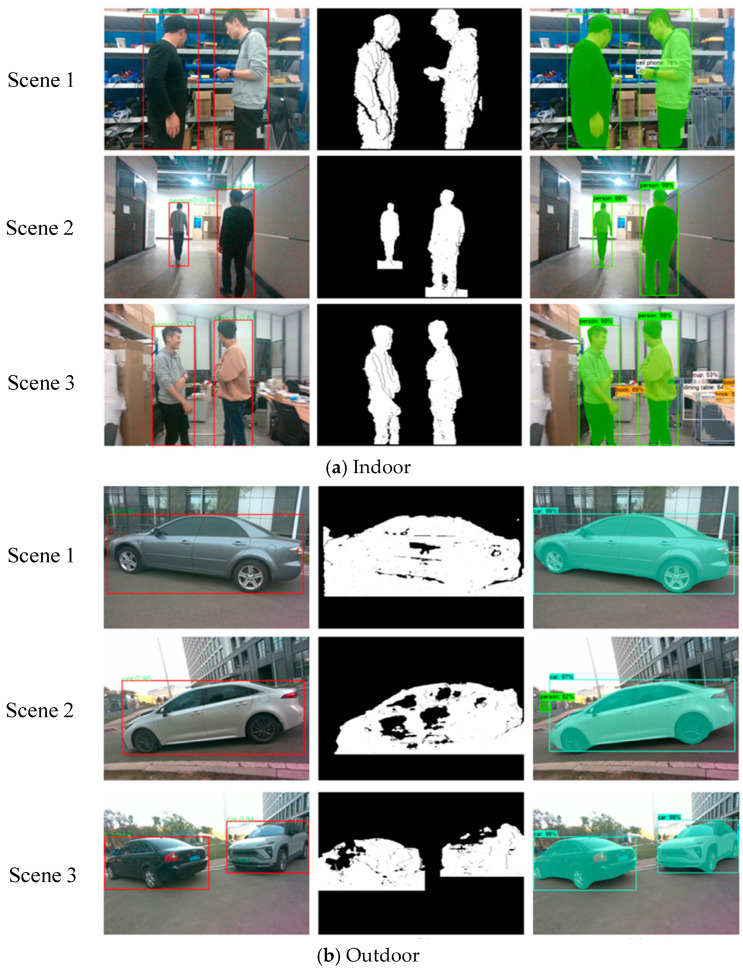
The result of different algorithms: in each subfigure, the left three images show the YOLO recognition results in different scenes, the middle image shows the mask results obtained by our algorithm, and the right image shows the segmentation results of Mask-RCNN. The red box represents the recognition bounding box of YOLO.

When testing the object segmentation algorithm, we used an AMD R7-5800H CPU and an NVIDIA RTX 3060 GPU. The image resolution was set to 640 × 480. The segmentation scenes are indoor and outdoor, as shown in Figure 8a,b, and the algorithm’s processing time is shown in Table 1.

Comparing the indoor scene images of Figure 8a, our algorithm can recognize the object’s outline, achieving similar results to Mask-RCNN. The average time consumption based on depth threshold segmentation is less than 1.5 ms, and the average time consumption of YOLO recognition is less than 20 ms. Meanwhile, the processing of the two is carried out in different computing units. The former is on the vehicle-mounted CPU, and the latter is on the vehicle-mounted GPU; that is, the processing of the two is in parallel. Since the time consumption of the YOLO algorithm is much less than that of Mask-RCNN, it can provide a picture processing capacity of 50 Hz, which is compatible with the current 30-Hz depth camera device. Also, the 2 ms time consumption of depth threshold segmentation meets the real-time requirement in the tracking module. Therefore, our algorithm can meet the real-time SLAM time requirements while achieving similar results.

For outdoor scenes Figure 8b, our algorithm obtained approximately target regions similar to Mask RCNN. However, due to the ground depth being similar to the target, the algorithm added some of the ground to the region. In addition, due to the lack of depth in some regions, the segmentation result of the algorithm had holes. However, these negative effects have little impact on SLAM, because these areas also have fewer features. The subsequent algorithm can still effectively separate the corresponding feature points of the target.

**Figure 9:**
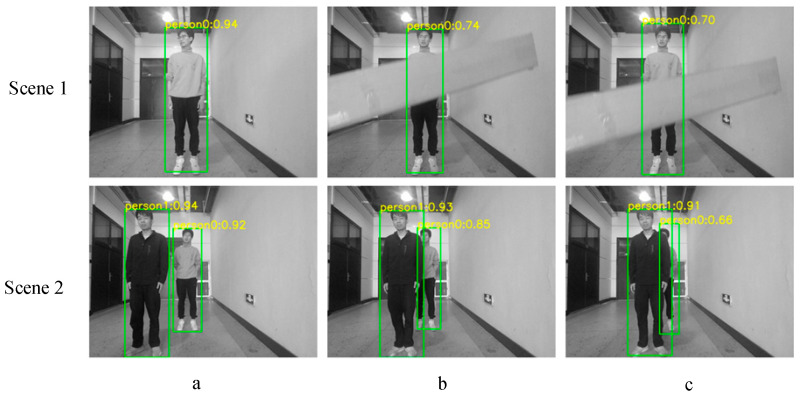
The different scenes for deep extraction algorithms.

To verify the effectiveness of the algorithm in dealing with occlusion, we conducted the experiment shown in Figure 9 and obtained the depth data shown in Table 2. Analyzing the depths detected by the algorithm and the actual depths in the table, we can see that our algorithm can still effectively obtain the depth of the object under occlusion, providing reliable data for segmentation and subsequent tracking.

### 5.2. Object Tracking

**Figure 10:**
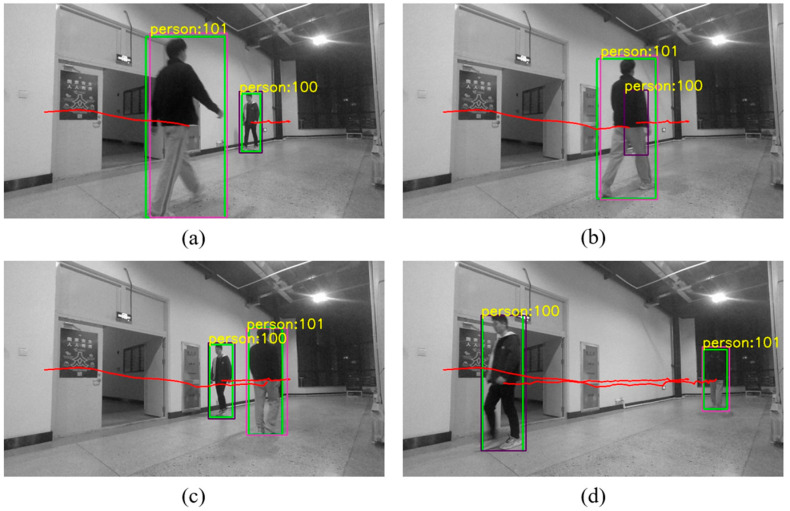
The result of our tracking algorithm: The green box represents the recognition box of YOLO, while the red and purple box represents the predicted box of our tracking algorithm. The number on the box indicates the ID of the person. The red line indicates the persons’ movement trajectory. (**a**) The stage during which the persons are not occluded. (**b**) The stage of mutual occlusion among persons. (**c**) The stage when the occlusion between persons has just ended. (**d**) The stage when the persons move away.

To verify the effectiveness of the tracking algorithm, we conducted an experiment as shown in Figure 10, which includes two dynamically moving characters. As shown in Figure 10b, there is mutual occlusion during the motion of the characters. In this case, recognition is interrupted; that is, the green box of character 100 in Figure 10b disappears. However, relying on predictive ability, the algorithm still correctly associates the character’s number when the character reappears in the position shown in Figure 10c, thus providing effective basis for subsequent target matching.

**Figure 11:**
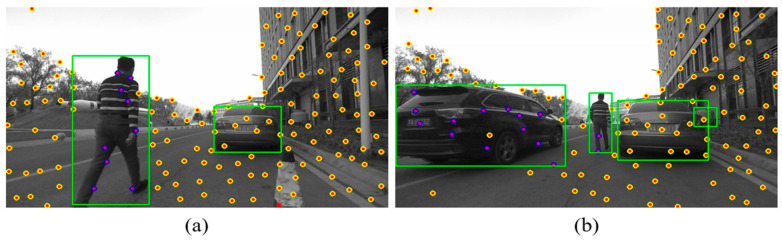
The classification result of features: The yellow circle represents static features, the purple circle represents dynamic features, and the green box is the YOLO detection box. (**a**) The scene where person enter the field of view. (**b**) The scene where car enter the field of view.

This algorithm mainly deals with dynamic objects, so relevant experiments on feature point classification of objects have also been carried out in this section, as shown in Figure 11. It can be seen from Figure 11 that the algorithm effectively marks the feature points on moving people and cars, while the points belonging to stationary car is classified as a static point.

### 5.3. Comparison of SLAM Robustness in Dynamic Environments

To evaluate our algorithm, we conducted comparative experiments on both public datasets and our own dataset, comparing VINS-mono, dynamic-VINS, and our algorithm. Among them, VINS-mono serves as the basic framework for the development of the algorithm in this paper, yet it lacks the capacity to handle dynamic environments. Dynamic-VINS is a relatively advanced open-source algorithm at present, but it only eliminates dynamic features. By comparing with VINS-mono, we can verify the ability of the algorithm proposed in this paper to deal with dynamic environments. Meanwhile, through comparison with the dynamic-VINS algorithm, we can verify the improvement in accuracy brought by the rigid-point-set modeling in our algorithm. The algorithm in this paper conducts experimental comparisons of three algorithms using three datasets. During the algorithm operation and evaluation process, everything is kept consistent except for the algorithms being verified.

**Figure 12:**
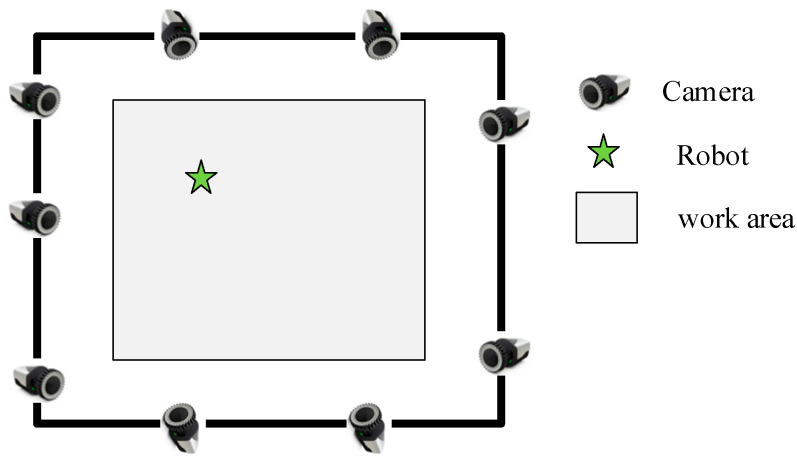
Schematic layout of the Motion Capture System.

**Figure 13:**
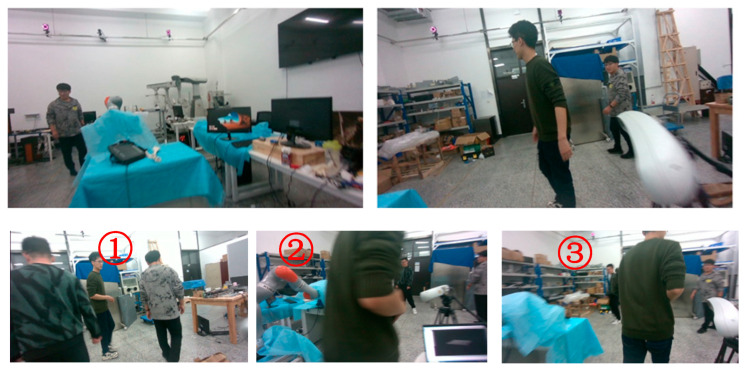
Schematic diagram of the indoor scene: The two pictures above are display diagrams of the scene. The following three pictures are three frames of data with short time intervals during the data collection process. Their order of appearance is consistent with the numerical labels.

The public dataset we selected is the market dataset in OpenLORIS-Scene. It is a dataset recorded in a supermarket, containing a large number of pedestrians and high dynamics. For indoor scenes, we use the Qualisys 3D Motion Capture Systems, which contains nine Miqus M3 arranged in rooms, as shown in Figure 12 to obtain the robot’s ground truth motion, which can provide sub-millimeter accuracy for the target trajectory. The Motion Capture Systems can support a maximum resolution of 4MP. In the full field of view high speed mode, it can support a frame rate of up to 650 frames per second, and the maximum capture distance reaches 18 m. The experimental site is an indoor room with a size of 5 m by 6 m. The acquisition device is a D455 equipped with target points recognizable by the motion capture system. The pose relationship between the target points and the camera is determined by the mechanical installation position. The D455 itself is equipped with an IMU component, and the pose between the two is calibrated using the kalibr method. Some of the scenes where data was collected are shown in Figure 13.

**Figure 14:**
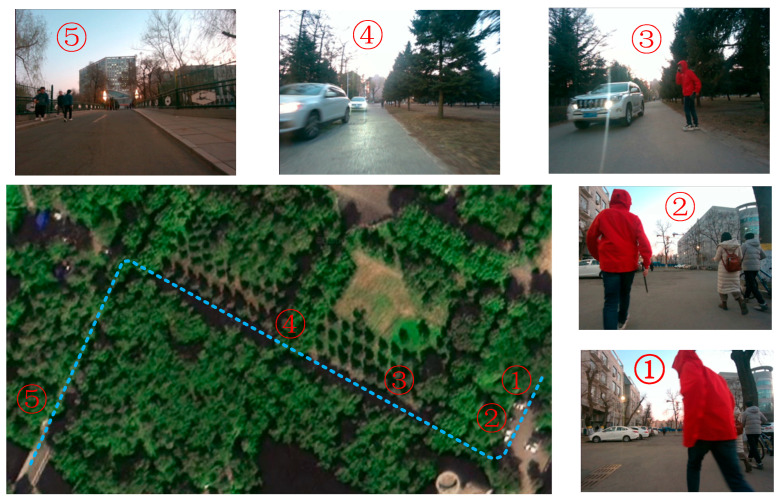
Schematic diagram of the outdoor scene trajectory. The large image at the lower left corner represents a top-view of the trajectory-collecting environment on campus. The blue dashed line indicates the motion schematic of the vehicle used. In the top-view, the scenes at the marked collection positions are shown as sub-images with corresponding numerical labels.

For outdoor scenes, we use differential GNSS (model M66H2H-Lite GNSS receiver, Johannes Kepler Luojia Technology Co., Ltd. in Wuhan, China) positioning devices to obtain the robot’s ground truth motion. This technology uses a fixed base station to collect satellite data. The base station sends the collected information to mobile devices via a 4G network. The mobile devices collect satellite data while receiving information from the base station and perform carrier-phase differential calculations to obtain high-precision positioning results. The positioning accuracy of the GNSS device in this paper can reach sub-centimeter level, and it can output 20H positioning information. The data collection platform is a mobile robot, as shown in Figure 7. The devices related to GNSS are shown in Figure 7b. The total length of the trajectory is 315 m. Some schematic diagrams of the scenes in the trajectory and route are shown in Figure 14.

The evaluation metrics we used are mainly Absolute Pose Error (APE) and Relative Pose Error (RPE), using EVO analysis tools [23]. Among them, APE is the error between the spatial position at each moment and the spatial position of the corresponding ground-truth trajectory at that moment, reflecting the overall degree of deviation. RPE is the error between the relative transformation of adjacent poses and that of the ground-truth trajectory, reflecting the local estimation accuracy. Based on the sufficiency of data, in the experiments of this paper, the evaluation metrics include the Root Mean Square Error (RMSE), Mean, Median, and Standard Deviation (STD) of ATE and RTE, respectively.

(22) $$\eta =\left(1-\frac{\beta }{\alpha }\right)\times 100\%$$

where η represents the degree of precision improvement, β represents the trajectory precision of the algorithm in this paper, α represents the trajectory precision of other algorithms.

**Figure 15:**
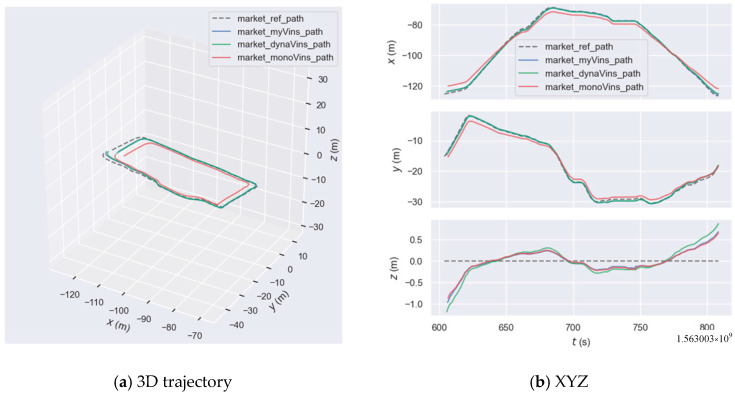
Camera trajectory graph in the market: the dashed line indicates the true path, the blue solid line indicates the trajectory generated by our algorithm, the green solid line indicates the trajectory generated by dynamic-VINS, and the red line indicates the trajectory generated by VINS-mono. The legend in the subsequent figure is the same as that in this figure.

Firstly, we conducted a comparative experiment in OpenLORIS-Scene, and the trajectories are shown in Figure 15a. The trajectory error results of the three algorithms shown in Figure 15b. The results of the statistics are presented in Table 3 and Table 4. As shown in Figure 15a, the algorithm proposed in this paper is overall closest to the true trajectory, followed by dynamic-VINS, and VINS-mono performs the worst. Analyzing Figure 15b, in the x- and y-axes, the algorithm in this paper does not have a significant advantage over dynamic-VINS, but both are significantly better than VINS-mono. In the *z*-axis direction, the algorithm in this paper does not have a significant advantage over VINS-mono, yet both are significantly better than dynamic-VINS. This is because VINS-mono does not have the ability to handle dynamic targets. Moving people and objects on the plane will seriously interfere with its horizontal positioning estimation. While dynamic-VINS uses a direct filtering method, which leads to a reduction in the features on people and objects used to constrain the *z*-axis, thus reducing the *z*-axis accuracy.

**Figure 16:**
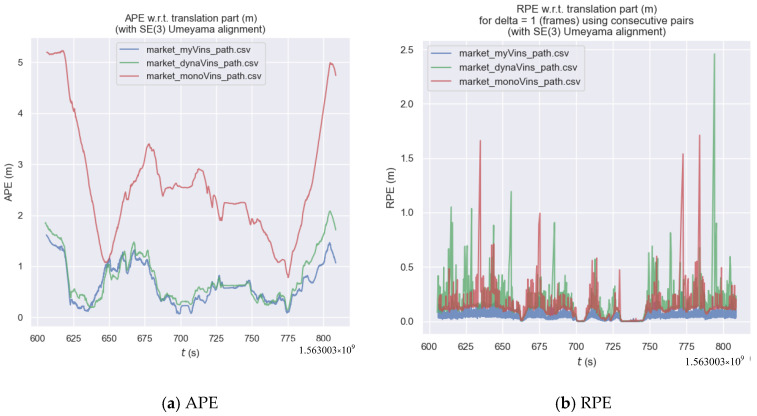
The graph of trajectory error in market.

By analyzing Figure 16, Table 3 and Table 4, we can see that: in terms of both APE and RPE indicators, the algorithm in this paper outperforms dynamic-VINS and is far superior to VINS-mono. Compared with dynamic-VINS, the algorithm in this paper has improvements of 13.6%, 12.3%, and 17.0% in the RMSE, MEAN, and STD indicators respectively. Compared with VINS-mono, the algorithm in this paper has improvements of 74.1%, 75.7%, and 66.6% in the RMSE, MEAN, and STD indicators, respectively. In summary, in the market scenario with high-dynamic targets, the algorithm in this paper is superior to the other two algorithms.

**Figure 17:**
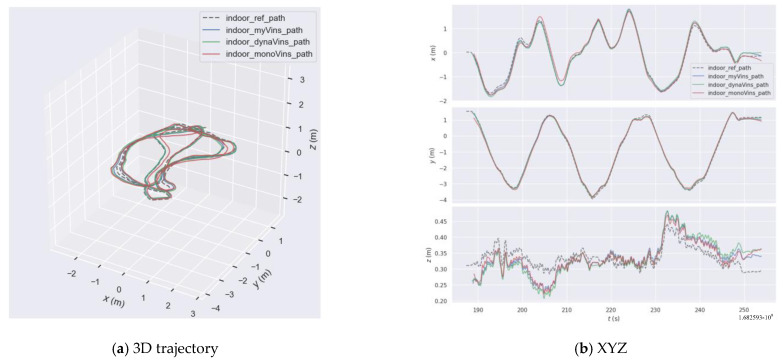
Indoor camera trajectory graph.

Next are the comparative experiments on our own indoor dataset. The trajectories are shown in Figure 17a. The results of the trajectory error of the three algorithms are shown in Figure 17b. The results of the statistics are shown in Table 5 and Table 6. In this part of the experiment, the camera repeatedly performed complex circular motions in a small scene. Relative to the camera, there were multiple instances where moving people in the scene occupied a large area. This experiment tests the algorithm’s ability to cope with interference caused by dynamic targets when the camera is in continuous rotational motion. Analyzing Figure 17, most of the trajectories of dynamic-VINS and the algorithm in this paper are close to the true trajectory, far superior to VINS-mono. Meanwhile, most of the trajectories of the algorithm in this paper show better results compared to dynamic-VINS.

**Figure 18:**
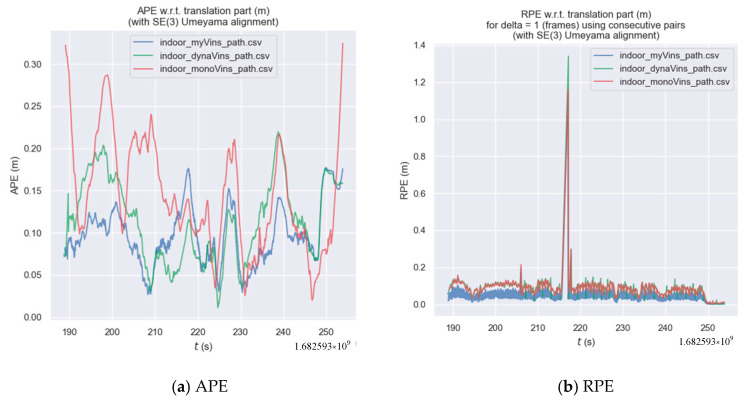
The graph of the indoor trajectory error.

By analyzing Figure 18, Table 5 and Table 6, we can see that: in terms of both APE and RPE indicators, the algorithm in this paper outperforms dynamic-VINS and is far superior to VINS-mono. Compared with dynamic-VINS, the algorithm in this paper has improvements of 17.3%, 15.3%, and 28.7% in the RMSE, MEAN, and STD indicators, respectively. Compared with VINS-mono, the algorithm in this paper has improvements of 35.0%, 32.7%, and 47.1% in the RMSE, MEAN, and STD indicators, respectively. In summary, in the market scenario with high-dynamic objects, the algorithm in this paper is superior to the other two algorithms.

**Figure 19:**
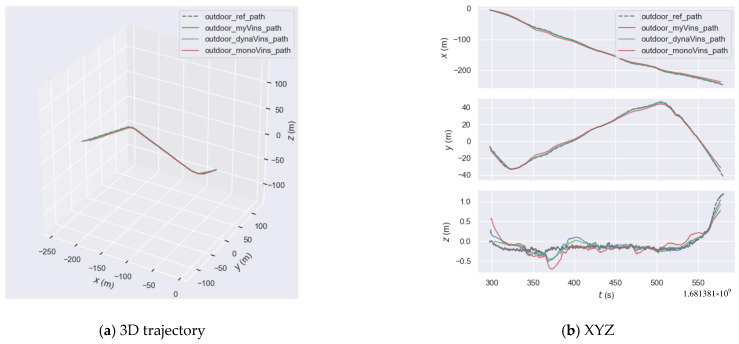
Outdoor camera trajectory graph.

Next are the comparative experiments on our own outdoor dataset. The trajectories are shown in Figure 19a. The results of the trajectory error of the three algorithms is shown in Figure 19b. The results of the statistics are shown in Table 7. In this part of the experiment, the camera has a wide field of view, which means the proportion of dynamic feature points is appropriate in most trajectories. This experiment tests the anti-interference ability of the algorithm proposed in this paper when the proportion of dynamics is low. By analyzing Figure 19, we can see that in several trajectory segments, such as the segments from 350 to 400 on the *x*-axis, from 350 to 400 and from 450 to 520 on the *y*-axis, and from 300 to 420 on the *z*-axis, the algorithm in this paper and dynamic-VINS are closer to the true trajectory and perform better than VINS-mono.

**Figure 20:**
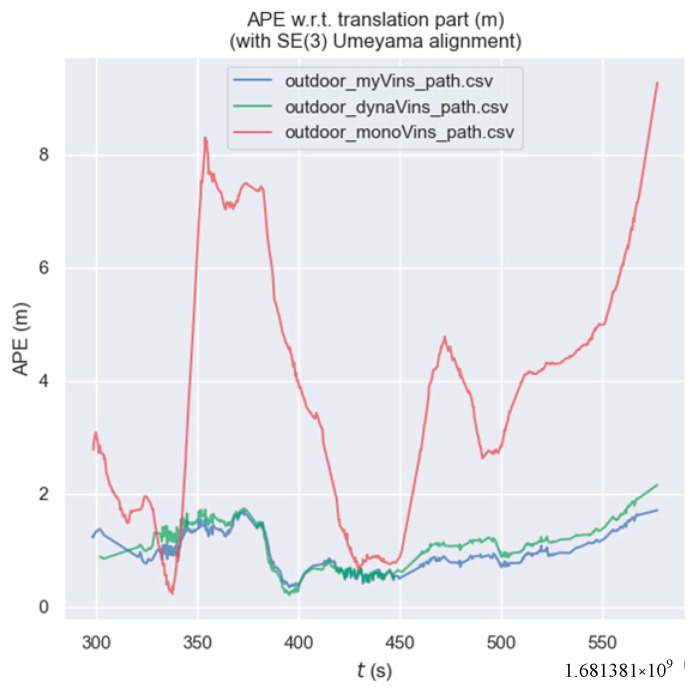
The graph of the outdoor trajectory error.

By analyzing Figure 20, Table 7, we can see that: in terms of both APE indicators, the algorithm in this paper outperforms dynamic-VINS and is far superior to VINS-mono. Compared with dynamic-VINS, the algorithm in this paper has improvements of 12.7%, 12.2%, and 16.7% in the RMSE, MEAN, and STD indicators, respectively. Compared with VINS-mono, the algorithm in this paper has improvements of 75.8%, 73.2%, and 84.8% in the RMSE, MEAN, and STD indicators, respectively. Since the ground-truth trajectory converted from differential GPS data does not contain attitude information, we do not compare relative error values.

In addition, as depicted in Figure 13 and Figure 14 of this paper, there are multi-target interactions within the self-collected datasets. In an indoor space, 2–3 dynamically moving individuals engage in multiple instances of cross-walking, creating complex scenarios where the device and people experience close-range occlusion. Similarly, in the outdoor datasets, there are numerous cases of people occluding one another, along with scenarios where vehicles pass by and obscure pedestrians. Thus, this algorithm is capable of addressing the issue of dynamic occlusion.

In summary, in a dynamic environment, the algorithm proposed in this paper improves the overall positioning accuracy by separating dynamic feature points and performing rigid-body modeling.

### 5.4. Runtime Comparison

To verify the real-time performance of the algorithm proposed in this paper, an analysis was carried out on the running time of the positioning system. We ran the algorithm proposed in this paper on the three-scene datasets mentioned above and obtained the average running time of each part of our algorithm. The hardware configuration is an AMD R7-5800H CPU and an NVIDIA RTX3060 GPU. The running times of the algorithms are shown in Table 8. The items marked with * in the table are three independent threads running in the system: the tracking thread, the state-estimation thread, and the target-detection thread. The item with the longest running time is state-estimation, which is 32.801 ms. Converted to speed, it is 30 frames per second, which well meets the real-time requirement.

### 5.5. Ablation Study of the Proposed Factors

**Figure 21:**
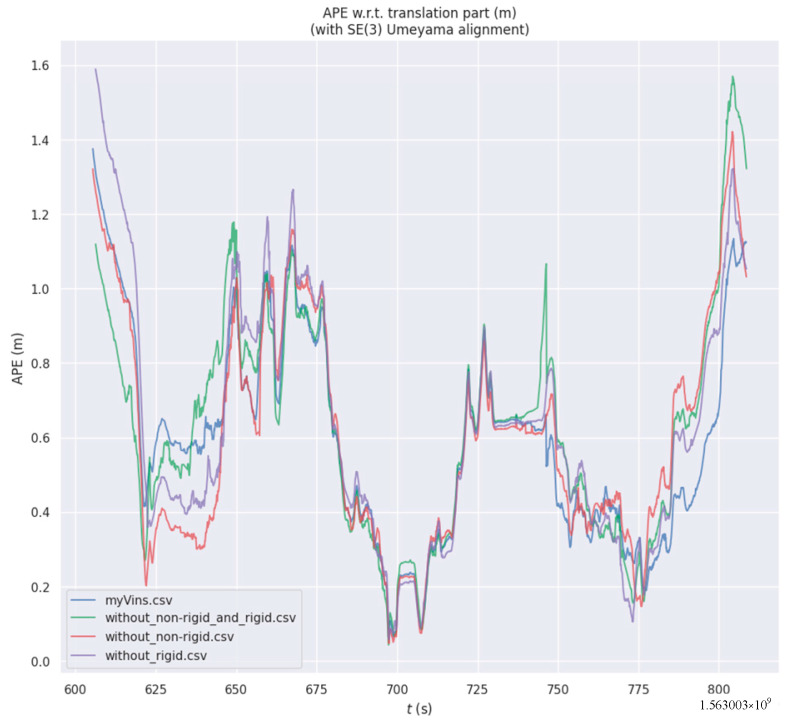
Trajectory errors under different factor graphs in the market.

To verify the effects of the independent components proposed in this paper, we conduct ablation experiments on the OpenLORIS-Scene dataset, including: without non-rigid factors, without rigid factors, without both, and with all factors. The experimental results are shown in Figure 21 and Table 9. We can observe that, compared to the case where none of the modules are enabled, enabling each module leads to an improvement in accuracy. Among them, in terms of the RMSE, MEAN, and STD metrics, the full-factors case has the highest accuracy. Regarding the MEDIAN metric, it is only lower than the “without rigid” case.

### 5.6. Robustness Analysis

**Figure 22:**
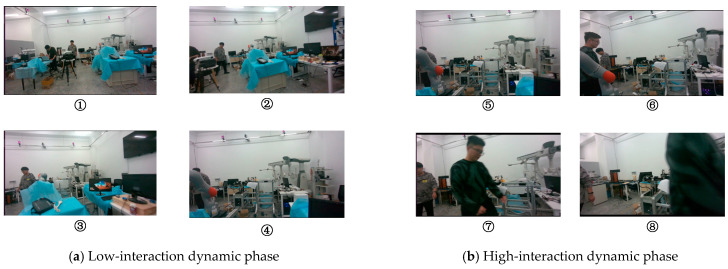
Process display diagrams for different stages. In the verification scenario, the visual images received by the device at different stages are presented in numerical order.

To verify the robustness of the algorithm proposed in this paper in occluded and interactive scenarios, a statistical analysis of the errors in different scene segments was carried out, as shown in Table 10. The low-interaction dynamic phase corresponds to Figure 22a, and the high-interaction dynamic phase corresponds to Figure 22b. Analyzing Table 10, we can conclude the following:

1. In both scenarios, the error of the algorithm in this paper is lower than that of dynamic-VINS. Specifically, in the low-interaction dynamic phase, the accuracy of the algorithm in this paper is improved by 25.9%, and in the high-interaction dynamic phase, the accuracy is improved by 37.7%.
2. Due to the involvement of highly interactive dynamic characters, the error of the algorithm in this paper increased by 25.0%, while the error of dynamic-VINS increased by 48.8%.
3. Therefore, we can conclude that our algorithm is more robust in dealing with highly interactive dynamics compared to dynamic-VINS.

## 6. Conclusions

This study proposes a dynamic SLAM algorithm based on factor graph optimization, which can effectively identify and track dynamic obstacles, improving the robustness of SLAM systems in dynamic environments. Experimental results in indoor and outdoor scenes show that the algorithm can achieve high localization accuracy and real-time performance. In addition, experiments on object segmentation and tracking, as well as the classification of moving object feature points, validate the effectiveness of the algorithm in these areas. Therefore, this study provides a new idea and method for the research of dynamic SLAM algorithms.

The disadvantage of this algorithm is that it may have some errors when dealing with outdoor scenes where the ground depth is similar to the target, and there is still room for improvement in the efficiency of the algorithm. Since the depth threshold segmentation algorithm is adopted in this paper, the operation of the system depends on a depth camera. The vehicle target estimated by the system is a family car. When facing a large-sized bus, due to the misestimation of its rigid body, the accuracy will decline. Future work can be done to improve and optimize in these areas, and the algorithm can be tested and evaluated in more datasets and scenarios to further validate its robustness and effectiveness.

## Figures and Tables

**Table 1:** The run times of algorithm (ms).

Scenes		Our Algorithm		Mask-RCNN
		YOLO	Segmentation	
indoor	Scenes 1	20.36	1.61	91.48
	Scenes 2	19.78	1.17	88.79
	Scenes 3	19.55	0.71	86.08
outdoor	Scenes 1	17.99	1.20	95.20
	Scenes 2	19.25	1.43	86.85
	Scenes 3	18.72	1.38	85.64

**Table 2:** The depth result from our algorithm (m).

Scenes		Our Algorithm		Truth	
		Person 1	Person 2		
Scene 1	a	2.004	-	2.00	-
	b	2.018	-		
	c	1.954	-		
Scene 2	a	2.899	1.847	2.800	1.900
	b	2.947	1.849		
	c	2.896	1.862		

The a indicates that the objects is not occluded, the b indicates that there is mild occlusion among the targets and the c indicates that there is severe occlusion among the targets.

**Table 3:** The APE of trajectory in market (m/frame).

e	Our SLAM	Dynamic-VINS	VINS-Mono
RMSE	**0.759**	0.878	2.931
MEAN	**0.654**	0.746	2.696
MEDIAN	**0.577**	0.621	2.514
STD	**0.384**	0.463	1.151

The bold-font represents the best result in the comparative experiment.

**Table 4:** The RPE of trajectory in market (m/frame).

e	Our SLAM	Dynamic-VINS	VINS-Mono
RMSE	**0.** **056**	0.193	0.170
MEAN	**0.** **046**	1.138	0.126
MEDIAN	**0.041**	0.125	0.116
STD	**0.033**	0.135	0.115

The bold-font represents the best result in the comparative experiment.

**Table 5:** The APE of indoor trajectory (m/frame).

e	Our SLAM	Dynamic-VINS	VINS-Mono
RMSE	**0.103**	0.124	0.158
MEAN	**0.097**	0.114	0.144
MEDIAN	**0.093**	0.115	0.132
STD	**0.035**	0.049	0.066

The bold-font represents the best result in the comparative experiment.

**Table 6:** The RPE of indoor trajectory (m/frame).

e	Our SLAM	Dynamic-VINS	VINS-Mono
RMSE	**0.** **057**	0.104	0.100
MEAN	**0.0** **43**	0.085	0.085
MEDIAN	**0.037**	0.089	0.088
STD	**0.038**	0.060	0.053

The bold-font represents the best result in the comparative experiment.

**Table 7:** The APE of outdoor trajectory (m/frame).

e	Our SLAM	Dynamic-VINS	VINS-Mono
RMSE	**1.024**	1.172	4.225
MEAN	**0.968**	1.102	3.613
MEDIAN	**0.920**	1.140	3.747
STD	**0.333**	0.400	2.192

The bold-font represents the best result in the comparative experiment.

**Table 8:** Results of runtime comparison (ms).

Feature Tracking	Feature Extraction	Tracking Thread *	Dynamic Feature Recognition	The Target State Estimation	State Estimation Thread *	Target Detection Thread *
1.536	1.178	5.644	0.625	0.112	32.801	19.680

* in the table are three independent threads running in the system.

**Table 9:** The APE of trajectory under different factor graphs in the market (m/frame).

e	Our SLAM	Without Non-Rigid	Without Rigid	Without Rigid & Non-Rigid
RMSE	**0.652**	0.6737	0.7016	0.7087
MEAN	**0.** **5912**	0.6065	0.6201	0.6406
MEDIAN	0.5863	0.6096	**0.5793**	0.6460
STD	**0.2742**	0.2932	0.3281	0.3033

The bold-font represents the best result in the comparative experiment.

**Table 10:** The APE of trajectory during the different stages (m/frame).

e	Low-Interaction Dynamic Phase		High-Interaction Dynamic Phase	
	Our SLAM	Dynamic-VINS	Our SLAM	Dynamic-VINS
RMSE	**0.0846**	0.1141	**0.1058**	0.1698
MEAN	**0.0843**	0.1132	**0.1051**	0.1689
MEDIAN	**0.0857**	0.1165	**0.1027**	0.1724
STD	**0.0071**	0.0143	**0.0121**	0.0176

The bold-font represents the best result in the comparative experiment.

## Data Availability

The original contributions presented in this study are included in the article. Further inquiries can be directed to the corresponding author.
